# Preliminary assessment of portable sleep monitoring for diagnosis of obstructive sleep apnea in children

**DOI:** 10.1007/s11325-023-02919-9

**Published:** 2023-09-18

**Authors:** Zhi-xiong Xian, Xin Wang, Yong-chao Chen, Yi-shu Teng

**Affiliations:** 1 Department of Otorhinolaryngology, Shenzhen Children’s Hospital, Shenzhen, Guangdong China; 2 Department of Otorhinolaryngology, Shenzhen Children’s Hospital, China Medical University, Shenzhen, Guangdong China

**Keywords:** Portable sleep monitor, Obstructive sleep apnea, Children, Diagnostic techniques and procedures

## Abstract

**Objective:**

By observing the differences in sleep parameters between portable sleep monitoring (PM) and polysomnography (PSG) in children, we aimed to investigate the diagnostic value and feasibility of PM in children with suspected obstructive sleep apnea (OSA).

**Study design:**

This prospective study enrolled consecutive children (aged 3–14 years) with suspected OSA in Shenzhen Children’s Hospital. They had PSG and PM in the sleep laboratory. Clinical parameters of the two sleep monitoring methods were compared.

**Results:**

A total of 58 children participated. They were classified into two groups according to age: 28 children aged 3 to 5 years and 30 children aged 6 to 14 years. No significant differences were observed in apnea-hypopnea index (AHI), lowest oxygen saturation (LSaO_2_), and mean oxygen saturation (MSaO_2_) between PM and PSG, but the sleep efficiency with PM was significantly higher (3–5 years age: 92.2 ± 11.3% vs 85.2 ± 14.3%, 6–14 years age: 93.2 ± 14.5% vs 84.8 ± 16.3%, both *P* < 0.05) than the sleep efficiency with PSG. Pearson correlation analysis indicated a strong correlation between AHI, LSaO_2_, MSaO_2_, and sleep efficiency measured by PSG and PM. Receiver operating characteristic curve (ROC) analysis showed that PM was a reliable diagnostic tool for OSA. PM has high sensitivity (3–5 years age: 95.8%, 6–14 years age: 96.3%) and low specificity (3–5 years age: 25.0%, 6–14 years age: 33.3%) for OSA in children. Thus, there is a low rate of missed diagnoses, but there is some inaccuracy in excluding children who do not have OSA.

**Conclusion:**

The results showed that PM has a good correlation with the various parameters of PSG. PM may be a reliable tool for diagnosing moderate and severe OSA in children, especially those who cannot cooperate with PSG or who have limited access to PSG.

## Introduction

Obstructive sleep apnea (OSA) is a common disease in children, with a prevalence of 1.2% to 5.7% [1]. Its symptoms include snoring, mouth breathing, apnea, frequent awakenings, bed-wetting, sweating, and hyperactivity. Occasionally, excessive daytime sleepiness may occur, which can seriously affect the child’s health and development, including growth retardation, cognitive and behavioral abnormalities, cardiovascular changes, and pulmonary hypertension [1, 2]. Enlargement of the adenoids and tonsils is the main cause of OSA in children, but persistent sleep-disordered breathing occurs in 33.7% of children after adenotonsillectomy [3, 4]. Therefore, accurate diagnosis and clear etiology are important to guide appropriate treatments.

Polysomnography (PSG) is the “gold standard” for diagnosing OSA and is the most commonly used method for diagnosing OSA. However, PSG has limitations, such as the need for expensive equipment and a sleep monitoring room, the need for professional staff, and the influence of multiple electrodes on the patient’s sleep. Some pediatric patients cannot cooperate with PSG, and patients must often wait a long time for a study, limiting its usefulness. In the USA, only about 10% of children undergo sleep apnea monitoring before undergoing adenoid and tonsil surgery, which means that many children have undergone surgical risks without an accurate diagnosis of OSA [5]. Practically, the diagnosis of OSA in children mostly relies on medical history and physical examination, while complaints of guardians and physical examinations such as adenoid tonsil size have no correlation with the diagnosis and severity of OSA in children [6–8]. Therefore, there is a need for a more economical and convenient diagnostic tool.

According to standards set by the American Academy of Sleep Medicine, sleep-related breathing tests are classified into four levels [9]. Portable sleep monitoring devices refer to types II-IV devices [9]. There are various types of sleep monitoring devices used clinically. In addition to the “gold standard” type I PSG, there are also type II portable monitoring (PM) devices, type III PM devices (such as the Stardust II, SOMNO medics), and Type IV PM devices (such as the Watch PAT system, micro-motion sensor sleep system, Morpheus Ox system, actigraphy, and pulse oximeter). Devices used for PM are small and lightweight. PM is a method of diagnosing sleep disorders by simplifying the biological electrodes. With the increasingly mature technology of PM devices, PM is gradually becoming more reliable for diagnosing OSA. In adults, studies have demonstrated agreement between a PSG and type III PMs for diagnosing OSA [10, 11]. However, such PM validation studies are scarce in pediatric patients. Validation studies based on adults cannot be extrapolated to children as the pathophysiology and management in children differ from those in adults [12]. This study aimed to analyze the parameters of PM and PSG in the same individuals to determine the value of PM in diagnosing OSA in children.

## Patients and methods

### Patients

This study included children who were admitted to the Otolaryngology Department of Shenzhen Children’s Hospital between March 2017 and January 2018. Consecutive children aged 3–14 years undergoing clinically indicated PSG to evaluate possible OSA were invited to participate with permission of parents or guardians. Children with obesity, Down syndrome, craniofacial malformations, sickle cell disease, neuromuscular disease, and mucopolysaccharide diseases were excluded. This prospective study was approved by the Ethics Committee of Shenzhen Childrens Hospital.

### Study design

According to a random number table, the subjects were assigned to have PM first or PSG first. They would complete PSG and PM on separate nights, the two tests occurring within 3 days. PSG and PM were conducted at Shenzhen Children’s Hospital Sleep Laboratory and were performed by sleep technicians with considerable experience in performing pediatric sleep studies.

PSG (SOMNO medics, V5) monitoring parameters included electroencephalogram (EEG) (8-lead), electronystagmogram, electrocardiogram (ECG), mouth and nasal airflow (thermistor and pressure monitoring), respiratory movement (thoracic and abdominal), finger blood oxygen saturation, postures, and mandible myoelectricity. The monitoring parameters of PM (SOMNO medics, Germany) included nasal airflow (pressure monitoring), finger blood oxygen saturation, and respiratory movement (thoracic and abdominal). Monitoring was conducted from 21:30 to 06:30 the next day. The original data were automatically recorded and analyzed, and then manually corrected.

All sleep scoring followed the guidelines of the AASM manual [9]. Apnea was defined as the cessation of airflow in the mouth and nose during sleep with thoracic and abdominal respiration. Hypopnea was defined as a ≥ 30% decrease in airflow signal amplitude lasting ≥ 10 s and accompanied by ≥ 3% oxygen desaturation. The time duration of respiratory events was defined as two or more respiratory cycles. PSG and PM parameters examined included the total sleep apnea–hypopnea index (AHI), mean oxygen saturation (MSaO_2_), lowest pulse oxygen saturation (LSaO_2_), and sleep efficiency. The diagnosis of OSA was based on the AHI, with categories of mild, moderate, and severe OSA defined by an AHI of 1 to 5, 6 to 10, and > 10 events/h, respectively [13]. 

### Statistical analysis

The statistical analysis in this study was conducted using SPSS version 26.0. All tests were two-tailed, with a significance level set at *α* = 0.05. A *P*-value less than 0.05 was considered statistically significant. The measurement data were presented as mean ± SD. AHI, LSaO_2_, MSaO_2_, and sleep efficiency compared by group using *t* test after testing for normal distribution. Correlation analysis was assessed using the Pearson correlation analysis. PSG results were used as the gold standard for diagnosing OSA. ROC curve analysis was performed to measure the area under the ROC curve (AUC). To further evaluate classification prediction, cross tabulation was used to calculate sensitivity and specificity.

## Results

### General data

A total of 58 children were enrolled. They were classified into two groups according to age: group 1 (3 to 5 years, 28 cases) and group 2 (6 to 14 years, 30 cases). Thirty-three children were males and 25 were females. Their average age was 7.3 ± 3.3 years. According to the reference for Chinese children and adolescents [8], no subject was overweight or obese.

### Comparison of diagnosis between PSG and PM

The manually scored PSG results were used as the gold standard. The comparison of diagnosis between PSG and PM is summarized in Table 1. In the 3–5 years age group, PSG diagnosed primary snoring (PS) in 3 children who were diagnosed with mild OSA by PM 1 child diagnosed with mild OSA by PSG was classified as PS by PM, 1 child diagnosed with mild OSA by PSG was classified as moderate OSA by PM, and 1 child diagnosed with moderate OSA by PSG was classified as severe OSA by PM, while diagnosis results were consistent for other children. In the 6–14 years age group, PSG diagnosed PS in 2 children who were diagnosed with mild OSA by PM, 1 child diagnosed with mild OSA by PSG was classified as PS by PM, and 1 child diagnosed with severe OSA by PSG was classified as moderate OSA by PM, and diagnosis results for the remaining children by the two methods were consistent.

**Table 1:** Comparison of diagnosis between PSG and PM

Monitoring style	Total	PS	OSA		
			Mild	Moderate	Severe
1. 3–5 years age					
PSG	28	4	11	6	7
PM	28	2	12	6	8
2. 6–14 years age					
PSG	30	3	12	5	10
PM	30	2	13	6	9

### Correlation between PSG parameters and PM parameters

There were no statistically significant differences in AHI, LSaO_2_, and MSaO_2_ between the two age groups (3–5 years and 6–14 years, *P* > 0.05), and the PM sleep efficiency was significantly higher (*P* < 0.05) than the PSG sleep efficiency, as indicated in Table 2. When comparing the AHI values between the two groups, the PM values were slightly higher than the PSG values, but this difference was not statistically significant (*P* > 0.05).

**Table 2:** Correlation between PSG parameters and PM parameters

Parameters	PSG	PM	*t*	*P*	r*	P*
1. 3–5 years age						
AHI (times/h)	9.6 ± 8.7	10.2 ± 5.6	− 0.329	0.743	0.885	< 0.001
LSaO_2_ (%)	84.2 ± 9.6	82.3 ± 10.3	0.717	0.476	0.898	< 0.001
MSaO_2_ (%)	91.6 ± 1.9	92.3 ± 2.3	− 1.201	0.235	0.684	< 0.001
Sleep efficiency (%)	85.2 ± 14.3	92.2 ± 11.3	− 2.034	0.047	0.482	0.009
2. 6–14 years age						
AHI (times/h)	10.2 ± 9.6	11.2 ± 10.5	− 0.400	0.690	0.872	< 0.001
LSaO_2_ (%)	84.4 ± 10.1	82.6 ± 9.4	0.722	0.473	0.862	< 0.001
MSaO_2_ (%)	91.6 ± 1.9	91.8 ± 2.1	− 0.369	0.714	0.721	< 0.001
Sleep efficiency (%)	84.8 ± 16.3	93.2 ± 14.5	− 2.110	0.039	0.523	0.003

*Pearson correlation analysis

Further Pearson correlation analysis was conducted on the AHI, LSaO_2_, MSaO_2_, and sleep efficiency of the two groups of children monitored by the two devices, and statistically significant differences were observed (*P* < 0.05). Both PM and PSG monitoring showed good correlations for various sleep parameters in both the 3–5 years and 6–14 years age groups. 

### Sensitivity and specificity of PM

**Fig. 1:**
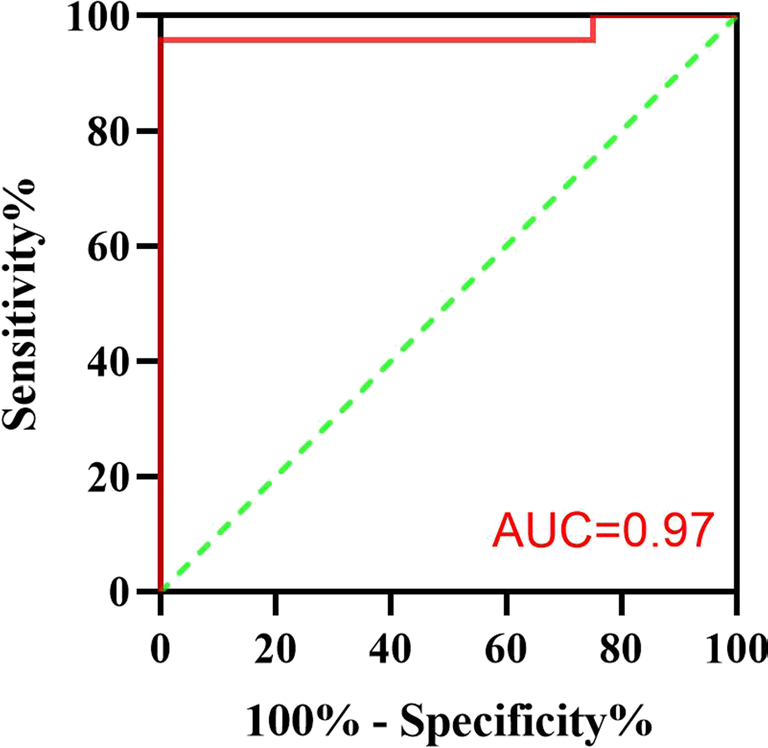
The ROC analysis of PM for diagnosing OSA in children aged 3–5 years using AHI-PSG ≥ 1 times/h

**Fig. 2:**
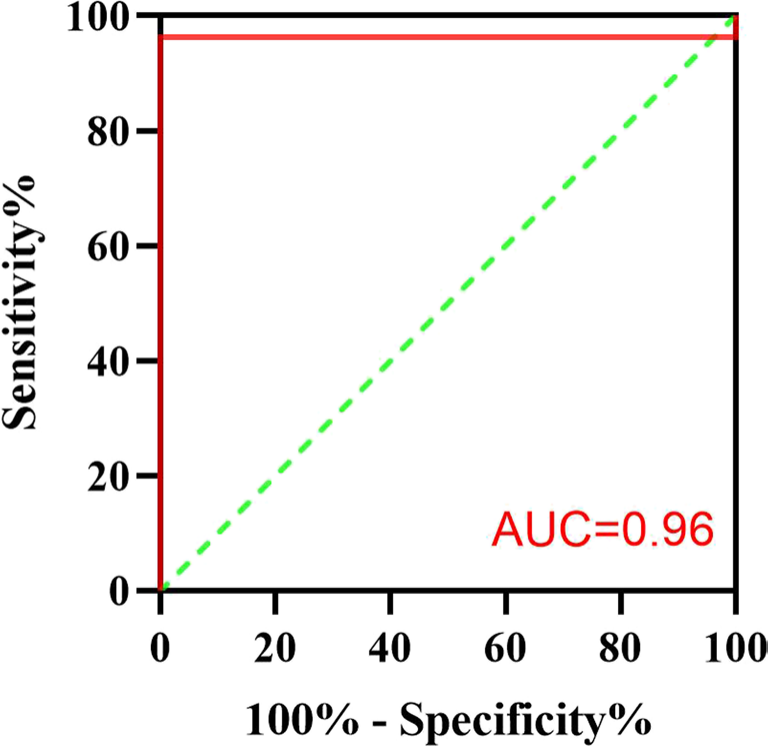
The ROC analysis of PM for diagnosing OSA in children aged 6–14 years using AHI-PSG ≥ 1 times/h

Using AHI-PSG ≥ 1 times/h as the diagnostic threshold for OSA in children, ROC analysis was performed to compare the diagnostic consistency of PM between the simple snoring group and the OSA group. The AUC for children aged 3–5 years was 0.97 (95% CI: 0.90–1.00, *P* = 0.003), with the sensitivity and specificity both at 95.8% and 25.0%, respectively, as shown in Fig. 1. Similarly, the AUC for children aged 6–14 years was 0.96 (95% CI: 0.89–1.00, *P* = 0.009), with the sensitivity and specificity both at 96.3% and 33.3%, respectively, as shown in Fig. 2. Results pertaining to classification prediction; ROC curve analyses and cross tabulation, are shown in Tables 3 and 4. These findings indicate that PM is a reliable predictor of OSA in children.

**Table 3:** Cross-tabulation results of PSG and PM using AHI ≥ 1, AHI > 5, and AHI > 10 times /h

Parameters	AHI ≥ 1	AHI > 5	AHI > 10
1. 3–5 years age			
AUC (95% CI)	0.97 (0.90–1.00)*	0.99 (0.96–1.00)*	1.00 (1.00–1.00)*
Sensitivity (%)	95.8%	100.0%	100.0%
Specificity (%)	25.0%	93.3%	95.2%
2. 6–14 years age			
AUC	0.96 (0.89–1.00)*	1.00 (1.00–1.00)*	1.00 (1.00–1.00)*
Sensitivity (%)	96.3%	100.0%	90.0%
Specificity (%)	33.3%	100.0%	100.0%

*Significant at *P* ≤ 0.05

**Table 4:** Cross-tabulation results of PSG and PM using mild, moderate, and severe sleep apnea definitions

Parameters	Mild	Moderate	Severe
1. 3–5 years age			
Sensitivity (%)	81.8%	83.3%	100.0%
Specificity (%)	82.4%	95.5%	95.2%
2. 6–14 years age			
Sensitivity (%)	91.7%	100.0%	90.0%
Specificity (%)	88.9%	96.0%	100.0%

## Discussion

The PM used in this study (SOMNO medics, Germany) measures nasal airflow (using a pressure sensor), finger pulse oximetry, and respiratory movement. The findings demonstrated a close agreement between PSG and PM in children. No significant differences were observed in AHI, LSaO_2_, and MSaO_2_ between PM and PSG, but the PM sleep efficiency was significantly higher than the PSG sleep efficiency. ROC analysis showed that PM was a reliable diagnostic tool for OSA, with PM having high sensitivity (3–5 years age: 95.8%, 6–14 years age: 96.3%) but low specificity (3–5 years age: 25.0%, 6–14 years age: 33.3%) for OSA in children. Thus, there was a low rate of missed diagnoses, but there was inaccuracy in excluding children without OSA. 

In recent years, studies have reported on the correlation of various monitoring parameters between PM and PSG and found no significant differences in the main parameters, suggesting that PM has a high sensitivity, specificity, and accuracy for the diagnosis of OSA in adults [14–16]. In fact, portable sleep monitoring has become a fundamental method for assessing sleep-disordered breathing in adults [16, 17]. However, the pathophysiology and management of OSA in children are not the same as that in adults and differs among various age groups. The sensitivity and specificity of the PM in diagnosing OSA in children has had large fluctuations due in part to the type of the PM used in different studies [18]. Tan et al. [19] used home sleep monitoring (respiratory polygraphy, RP) in 100 pediatric patients with suspected OSA and applied it with simultaneous PSG. Their findings, when using PSG-AHI ≥ 5 times/h, revealed a sensitivity of 62.5%, specificity of 100%, positive predictive value of 100%, negative predictive value of 80%, and an AUC of 0.81, and these results indicated a strong correlation between the AHI values derived from both methods. Weimin et al. [20] observed a significant positive correlation between WatchPAT-AHI and PSG-AHI (Pearson’s correlation coefficient, *r* = 0.92,* P* < 0.001) [18]. In a comparative study by Cheung et al. [21] involving 45 pediatric patients suspected OSA, RP demonstrated a strong correlation with PSG-derived AHI (*r* = 0.98, *P* < 0.001). Masoud et al. [22] used MediByte in 70 pediatric participants (median age 10.8 years) and applied it with simultaneous PSG. They found that for an AHI cutoff of ≥ 5 times/h, the AUC was 0.89, the sensitivity was 95.5%, and the specificity was 66.7%; for an AHI cutoff of ≥ 10 times/h, the AUC was 1.00, the sensitivity was 100.0%, and the specificity was 93.4%. Lesser et al. [23], in a study involving 25 obese children with an average age of 13.6 years, utilized Apnealink in conjunction with PSG monitoring. They found that for an AHI cutoff of ≥ 5 times/h, Apnealink exhibited a sensitivity of 85.7% and specificity of 83.3%.

AHI is the main index for assessing the severity of OSA. The literature shows conflicting results with some studies showing PM overestimating AHI [12, 23], whereas others show PM underestimating AHI [24, 25]. However, in this study, the PM-AHI value was generally higher than the PSG-AHI value. Due to the lack of EEG monitoring with PM, changes in the sleep structure cannot be observed, and fluctuations in the respiratory rate during sleep may be mistakenly identified as wakefulness, resulting in a reduced calculation of sleep efficiency and an increase in the number of apnea or hypopnea events per hour. At the same time, false positives may be generated from limb movements during sleep. Underestimation of the AHI may also occur due to the number of apneas being divided by total recorded time rather than total sleep time [24, 25]. Therefore, the use of a nighttime sleep log may assist in the interpretation of sleep–wake periods measured by PM. This will likely increase the validity and reproducibility of portable devices.

Each diagnostic modality clearly has its own set of advantages and disadvantages. PSG requires the establishment of a sleep laboratory with professional personnel on night duty with inherent costs. Due to time constraints, limited funds, equipment, and personnel, PSG has not met the clinical demands. Furthermore, for children, PSG with leads and electrode stickers causes disruption of sleep, and poor cooperation from pediatric patients makes large-scale screening impossible. However, PSG may also provide additional valuable information. The inclusion of EEG data in PSG recordings enables the assessment of various aspects of sleep and neurological activity. This data can help in characterizing different sleep stages, detecting abnormal brain wave patterns, and diagnosing sleep disorders such as sleep apnea, insomnia, and narcolepsy. Therefore, PSG may be a good choice for patients with significant comorbid medical conditions that may degrade the accuracy of PM and patients suspected of having comorbid sleep disorders. PM is a convenient, low-cost, and easy-to-use tool with high sensitivity for diagnosing OSA in children, but there is some inaccuracy in identifying children who do not have OSA. It is also important to consider other factors regarding the diagnosis of OSA, such as symptoms, other test results, and risk factors, when interpreting results of sleep tests [18].

This study has several evident limitations. First, the small sample size undermines the representativeness of the findings. The participants consisted solely of children referred to a sleep clinic for investigation of OSA who were otherwise healthy. Second, the study exclusively assessed the use of PM in an inpatient setting without validating its applicability in a home environment. Third, the study relied on single-night sleep monitoring rather than continuous monitoring over multiple nights. This approach may potentially result in missing positive cases or misclassifying disease groups due to confounding factors such as the first-night effect. Fourth, the PM employed in this study lacked the capability to differentiate EEG data and perform sleep staging. Consequently, it may lead to underdiagnosis of other sleep disorder-related illnesses.

In summary, the PM accurately identifies OSA when tested in a sleep laboratory on pediatric patients with suspected OSA referred for symptoms of sleep-related breathing disorder. The PM-AHI strongly correlated with the PSG-AHI. And the sensitivity for detection of moderate and severe OSAHS diagnosed by PSG using PM was very high. PM may play an important role in diagnosing moderate and severe OSA, especially in patients who cannot cooperate with PSG or who have limited access to PSG.

## Data Availability

The datasets used and/or analyzed during the current study are available from the corresponding author on reasonable request.
